# Liver Transplantation With Incidental Gallbladder Carcinoma in the Recipient Hepatectomy

**DOI:** 10.1155/1994/76803

**Published:** 1994

**Authors:** W. Kennethwashburn, W. Davidlewis, Roger L. Jenkins

**Affiliations:** 1 The Division of Liver Transplantation and Hepatobiliary Surgery New England Deaconess Hospital Boston MA USA; 2 Division of Liver Transplantation and Hepatobiliary Surgery New England Deaconess Hospital 110 Francis St. Suite 8 C Boston MA 02215 USA

## Abstract

Gallbladder carcinoma(GBCA) is the most common malignancy of the biliary tract^1^ and is often found seredipitiously after cholecystectomy. We report the first two cases of incidental GBCA in the native gallbladder of two liver transplant recipients. Both patients are 2.5 years following uneventful orthotopiic liver transplantation(OLTx) with no evidence of recurrent disease. Pathology of both recipients was early and favorable. Neither patient received any further therapy. Given the incidence of GBCA and the evolution of OLTx we would anticipate this finding to be more prevalent.


*HPB Surgery*, 1994, Vol. 8, pp. 147–149
Reprints available directly from the publisher
Photocopying permitted by license only

© 1994 Harwood Academic Publishers GmbH
Printed in Malaysia

### Liver Transplantation with Incidental Gallbladder Carcinoma in the Recipient Hepatectomy

W. KENNETH WASHBURN, W. DAVID LEWIS and ROGER L. JENKINS

The Division of Liver Transplantation and Hepatobiliary Surgery, New England Deaconess Hospital, Boston, MA

Gallbladder carcinoma(GBCA) is the most common malignancy of the biliary tract[1] and is often found seredipitiously after cholecystectomy. We report the first two cases of incidental GBCA in the native gallbladder of two liver transplant recipients. Both patients are 2.5 years following uneventful orthotopiic liver transplantation(OLTx) with no evidence of recurrent disease. Pathology of both recipients was early and favorable. Neither patient received any further therapy. Given the incidence of GBCA and the evolution of OLTx we would anticipate this finding to be more prevalent.

KEY WORDS: Liver transplant Gallbladder neoplasms

### INTRODUCTION

Surgeons performing biliary tract surgery will encounter GBCA 1–2% of the time.[2] Most long term survivors of GBCA had incidental discovery of the malignancy, with cholecystectomy as the only surgical therapy.[3–6] Though some authors favor aggressive resection for early GBCA,[7–10] most agree that there is little role for curative surgery in the more advanced stages of this disease. OLTx has assumed a much more limited if not non-existant role in the treatment of biliary tract malignancies, most notably cholangiocarcinoma. Recurrence rates are extraordinarily high and most patients succumb to recurrent disease. Five year survival in most series is at best 20%.[11] OLTx has not been a treatment modality for GBCA because of the advance stage of disease in which most patients present and the propensity for local lymphatic spread.[12] However, given that GBCA is found incidently in 1–2% of cases of biliary tract surgery,[13] this finding should occur with a similar frequency during OLTx. To date, we believe there have been no reported cases of incidental GBCA in the recipient hepatectomy specimen. We report here two patients who underwent OLTx who had incidental findings of GBCA in their native liver.

### CASE REPORT

The first patient is a 53 year old female who underwent an uneventfull OLTx on 8/11/91 for primary biliary cirrhosis. She is maintained on FK506–PRED. There was no gross abnormality of her native gallbladder noted at surgery. Her pathology revealed the native gallbladder to have extensive adenomatous hyperplasia with foci of well differentiated adenocarcinoma($T_1N_0M_0$, AJCC stage 1). There was no muscular invasion and all lymph nodes were negative for metastatic disease. There were 15 stones measuring 1.0 cm in diameter found in the specimen. Her post–operative course was uneventful. She remains well with normal graft function and no evidence of disease 2.5 years following her transplant.

The second patient is a 65 year old male who underwent OLTx on 7/6/91 for primary sclerosing cholangitis. He is maintained on FK506-PRED. Pre-operative ultrasound revealed the gallbladder to be thickened but no evidence of any mass lesions. There were no abnormalities noted at the time of surgery. There was a papillary growth at the fundus of the gallbladder noted on pathologic examination which revealed adenocarcinoma with extension in

*Address for correspondence:* Roger L. Jenkins, MD. Division of Liver Transplantation and Hepatobiliary Surgery, New England Deaconess Hospital, 110 Francis St. Suite 8 C, Boston, MA 02215 (617)-632-9779, (617)-632-7555 FAX

the advential soft tissue and no evidence of lymph node involvement($T_2N_0M_0$, AJCC stage 11). There was no evidence of gallstones. He remains well 2.5 years following transplantation with no evidence of disease.

### DISCUSSION

GBCA remains one of the most fatal malignancies of the Gl tract with 5 year survival in most series only 5–10%. The incidence of GBCA has been calculated to be 2.5 per 100,00 inhabitants.[19] This incidence is increased to 1–2% in patients undergoing biliary tract surgery.[2,19,20] The peak incidence is usually in the seventh decade of life.[21] Cholelithiasis is found in 60–80% of cases of GBCA,[22] though no direct causative effect has yet been identified. Histologically, adenocarcinoma is the most common type with papillary carcinoma having the most favorable prognosis. Lymphatic and/or vascular invasion are indicative of a less favorable outcome. Surgical treatment is often palliative as most cases of GBCA at presentation are beyond the scope of a curative resection. Those patients who did enjoy long term survival often had the malignancy discovered incidently at the time of cholecystectomy.[4,6,17,18] Though this scenario is infrequent(1% of all CCY), controversy continues to exist with regards to the treatment of those patients with incidental GBCA. Cholecystectomy alone is often considered adequate surgical management for non-invasive $T_1$ GBCA.[13-16] Radical resection for more advanced node negative disease has been advocated by Shirai *et al.*[8] They perform a wedge resection of the gallbladder bed with lymph node dissection and excision of the supraduodenal extra-hepatic biliary tree for $T_2$ lesions(invades perimuscular connective tissue with no extension beyond the serosa). With this approach they have extended the 5 year survival from 40% to 90%. They found no survival advantage to $T_1$ lesions (confined to the mucosa) treated with radical resection.

OLTx has become a successful treatment for patients with end-stage liver disease with one year survival in most programs 75–80%. Reports of OLTx for most malignancies including cholangiocarcinoma and large primary hepatomas have universely shown extremely poor survival with extraordinarily high recurrence rates and most patients succumbing to their native disease.[11,23-25] Five year survival for patients transplanted for cholangiocarcinoma is just 17%.[11] There are no reports of OLTx for primary GBCA, nor are there any reports of incidental findings of GBCA in the recipient hepatectomy specimen. Given the generally dismal prognosis for GBCA when it is not incidentally found, it is not surprising that OLTx is not a therapeutic option for patients with this disease. However, given the incidence of GBCA found in patients undergoing biliary tract surgery, one would anticipate the finding of incidental GBCA to be somewhat similar in patients undergoing OLTx. This incidence will likely be somewhat less considering the pediatric population and the younger age of most OLTx patients compared with the peak incidence of GBCA in the seventh decade of life in the general population.

The dissection for OLTx does not encompass the nodal drainage of the gallbladder nor does it routinely result in excision of the extra-hepatic biliary tree as is done in the radical re-resection advocated by Shirai.[7] As an incidental finding in the recipient gallbladder, these patients can be observed without other intervention. Neoadjuvant therapy has been extremely disappointing with minimal response rates.[26] Without evidence of more extensive disease ($T_2$) there is no need to consider further surgical intervention.

We report the first two cases of incidental GBCA found in the recipient hepatectomy specimen of patients undergoing OLTx. Both patients remain alive and well with NED 2.5 years after transplantation. Given the frequency of GBCA and OLTx we expect this entity to be more common. With early lesions, as most incidental GBCA are, little else need be done besides the routine follow-up. If the incidental GBCA is of a more advanced stage then one should consider re-resection of the biliary tree and regional lymph node dissection. However, liver transplantation is not a therapeutic option for incidental GBCA found in the non-transplant patient.

### REFERENCES

1. Holmes, H. L. and Mark, B. D. (1971) Carcinoma of the Gallbladder. *Surg. Gynecol. Obstet.*, **133**, 561–564.
2. Jones, R. S. (1990) Carcinoma of the Gallbladder. *Surg. Clin. North. Am.*, **70**, 1419–1428.
3. Fahim, R. B., Ferris, D. O. and McDonald, J. R. (1963) Carcinoma of the Gallbladder: an appraisal of its surgical treatment. *Arch. Surg.*, **86**, 334–341.
4. Appleman, R.M., Morlock, C. G., Dahlin. and D. C., Adson, M. A. (1963) Long term survival in carcinoma of the gallbladder. *Surg. Gynecol. Obstet.*, **117**, 459–464.
5. Nevin, J. E., Moran, T. J., Kay, S. and King, R. (1976) Carcinoma of the gallbladder: staging, treatment, and prognosis. *Cancer*, **37**, 141–148.
6. Bergdahl, I. (1980) Gallbladder carcinoma first diagnosed at microscopic examination of gallbladder removed for pre-sumed benign disease. *Ann. Surg.*, **191**, 19–22.
7. Shirai, Y., Yoshida, K., Tsukada, K., Muto, T. and Wantanbe, H. (1992) Radical Surgery for Gallbladder Carcinoma. *Ann. Surg.*, **216**, 565–568.
8. Shirai, Y., Yoshida, K., Tsukada, K. and Muto, T. (1992) Inapparent Carcinoma of the Gallbladder. *Ann. Surg.*, **215**, 326–331.
9. Torterolo, E., Aizen, B., Silva, C., Bergalli, I. Misa, C. and

Beltran, R. (1993) An Approach to Histologically Diagnosed gallbladder Carcinoma following Cholecystectomy for presumed benign disease. *Jour. Surg. Onc.-Supp.*, **3**, 175–178.

10. De-Aretxabala, X., Roa, I., Araya, J. C., Fonseca, L., Wistuba, I. and Flores, P. (1992) Gallbladder cancer in Chile. A report on 54 potentially resectable tumors. *Cancer*, **69**, 60–65.
11. Penn, I. (1991) Hepatic Transplantation for Primary and Metastatic Cancers of the Liver. *Surgery*, **110**, 726–735.
12. Fahim, R. B., McDonald, J.R., Richards, J. C. and Ferris, D. O. (1962) Carcinoma of the Gallbladder: A Study of its Modes of Spread. *Ann. Surg.*, **156**, 114–123.
13. Piehler, J. M. and Crichlow, R. W. (1978) Primary Carcinoma of the Gallbladder. *Surg. Gynecol. Obstet.*, **147**, 929–942.
14. Adson, M. A. (1973) Carcinoma of the Gallbladder. *Surg. Clin. North. Am.*, **53**, 15.
15. Bivins, B. A., Meeker, W. A., Weiss, Jr., D. L. and Griffen, W. O., Jr. (1975) Carcinoma *in situ* of the Gallbladder: a dilema. *South. Med. J.*, **68**, 297–300.
16. Albores-Saavedra, J., Angeles-Angeles, A., Manrique, J. J. and Henson, D. E. (1984) Carcinoma *in situ* of the gallbladder: a clinicopathologic study of 18 cases. *Am. J. Surg. Pathol.*, **8**, 323–333.
17. Frank, S. A. and Spjut, H. J. (1967) Inapparent Carcinoma of the Gallbladder. *Am. Surg.*, **33**, 367–372.
18. Kott, I. and Urca, I. (1974) Carcinoma *in situ* of the gallbladder and routine cholecystectomy. *Med. Chir. Dig.*, **3**, 181–182.
19. Burdette, W. J. (1957) Cancer of the gallbladder. *Ann. Surg.*, **145**, 832–837.
20. Thorbjarnarson, B. and Glenn, F. (1959) Carcinoma of the gallbladder. *Cancer*, **12**, 1009–1015.
21 DoCarmo, M., Perpetuo, M. D. and Valdivieso, M. D., *et al.* (1978) Natural history study of gallbladder cancer; a review of 36 years experience at the MD Anderson Hospital and Tumor Institute. *Cancer*, **42**, 330–335.
22. Silk, Y. N., Douglass, H.O., Nava, Jr., Driscoll, H. R., D. L. and Tratarian, G. (1989) Carcinoma of the gallbladder. *Ann. Surg.*, **210**, 751–757.
23. Jenkins, R. L., Pinson, C. W. and Stone, M. D. (1989) Experience with transplantation in the treatment of liver cancer. *Cancer Chemother. Pharmacol.*, **23**, S104.
24. Ringe, B., Wittekind, C. and Bechstein, W. O., *et al.* (1989) The role of liver transplantation in hepatobiliary malignancy. *Ann. Surg.*, **209**, 88–98.
25. Stiber, A. C., Marino, I. R. and Iwatsuki, S., *et al.* (1989) Cholangiocarcinoma in sclerosing cholangitis: The role of liver transplantation. *Int. Surg.*, **74**, 1–10.
26. Eastern Cooperative Oncology Group report: chemotherapy of gallbladder, bile duct, and primary liver cancer: more bad news. (1984) *Cancer*, **54**, 965–969.

